# Evaluating sex as a biological variable in preclinical research: the devil in the details

**DOI:** 10.1186/s13293-016-0066-x

**Published:** 2016-02-11

**Authors:** Cara Tannenbaum, Jaclyn M. Schwarz, Janine A. Clayton, Geert J. de Vries, Casey Sullivan

**Affiliations:** Institute of Gender and Health, The Canadian Institutes of Health Research, Montreal, Canada; Psychological and Brain Sciences, University of Delaware, Newark, DE USA; U.S. National Institutes of Health Office of Research on Women’s Health, Bethesda, MD USA; Neuroscience Institute, Georgia State University, Atlanta, GA USA; Organization for the Study of Sex Differences, USA

**Keywords:** Sex as a biological variable (SABV), Peer review, Evaluation criteria, CIHR, NIH policy

## Abstract

Translating policy into action is a complex task, with much debate surrounding the process whereby US and Canadian health funding agencies intend to integrate sex and gender science as an integral component of methodological rigor and reporting in health research. Effective January 25, 2016, the US National Institutes of Health implemented a policy that expects scientists to account for the possible role of sex as a biological variable (SABV) in vertebrate animal and human studies. Applicants for NIH-funded research and career development awards will be asked to explain how they plan to factor consideration of SABV into their research design, analysis, and reporting; strong justification will be required for proposing single-sex studies. The Canadian Institutes of Health Research is revising their peer review accreditation process to ensure that peer reviewers are skilled in applying a critical lens to protocols that should be incorporating sex and gender science. The current paper outlines the components that peer reviewers in North America will be asked to assess when considering whether SABV is appropriately integrated into research designs, analyses, and reporting. Consensus argues against narrowly defining rules of engagement in applying SABV, with criteria provided for reviewers as guidance only. Scores will not be given for each criterion; applications will be judged on the overall merit of scientific innovation, rigor, reproducibility, and potential impact.

## Background

In May 2014, the US National Institutes of Health announced a policy aimed at integrating sex as a biological variable (SABV) into biomedical research [1]. The announcement led to a spirited debate in the scientific literature and popular press on the subject of promoting due diligence in exploring sex differences while respecting the principles of scientific freedom [2–5]. Our objective here is to describe the decisions taken by two major North American health funding agencies, the US National Institutes of Health (NIH) and the Canadian Institutes of Health Research (CIHR), for applying metrics to evaluate SABV by peer reviewers in their respective organizations [1, 6].

## Main text

In order to determine the preferred scoring method to gauge the appropriateness of SABV in research protocols involving preclinical studies with vertebrate animal and humans, an iterative process was undertaken by both the NIH and CIHR. The NIH’s Office of Research on Women’s Health championed the process in the USA, with SABV being considered as only one element of a broad NIH initiative to enhance rigor, transparency, and reproducibility of preclinical biomedical research [7]. The Institute of Gender and Health championed SABV on behalf of the CIHR, as part of the Canadian federal government’s Health Portfolio policy on sex-and-gender-based analysis [8]. Two independent champions, two different policies, yet the selection of metrics for the evaluation of SABV ended up being remarkably similar for both funding agencies. The process consisted of (a) an environmental scan of the literature for critical components of SABV; (b) key informant consultations and stakeholder forums; and (c) high-level discussions by decision-makers inside each funding organization. In February 2015, the US Office of Research on Women’s Health and Canada’s Institute of Gender and Health compared notes. They then created a consensus list of 13 evaluation criteria that might be used as a minimal standard for peer reviewers evaluating SABV information.

**Fig. 1:**
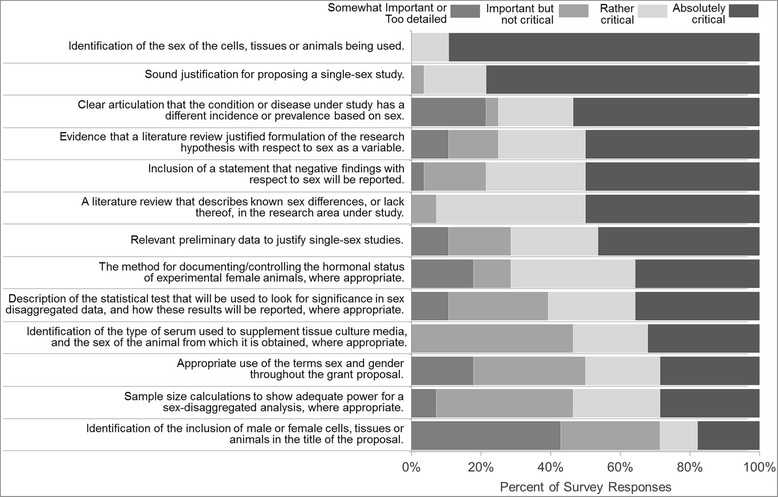
Ranking of the 13 SABV evaluation criteria according to the respondents’ assessment of absolutely critical or rather critical

As part of the feedback process on the evaluation criteria, the Organization for the Study of Sex Differences (OSSD) anonymously surveyed health researchers who had registered for its 2015 annual meeting on their perceptions of the importance of including each of these elements in a peer review rating scale for SABV. Figure 1 illustrates the proportion of online survey respondents (*n* = 30) who assessed each of the 13 evaluation criteria as being absolutely critical, rather critical, important but not critical, or somewhat important/not critical in the assessment of SABV. Seventy-five researchers then participated in a stakeholder’s forum held at the OSSD meeting on April 21, 2015, in Palo Alto, CA, to discuss and vote on issues that generated the most diverse responses in the initial survey. Participants were international, originating from Canada, South America, Europe, and Australia, with a majority representation from the USA. Almost three quarters of respondents considered themselves well versed in the study of sex differences. Sixty-seven percent stated they studied sex differences for 6 years or more. The distribution of responses shown in Fig. 1 is found to be representative of the response of the larger group of 75 people at the stakeholder’s forum.

The majority of participants proposed that peer reviewers should use the evaluation criteria as a SABV checklist guide only and that individual scores not be assigned to each criterion. Respondents expressed a strong preference for incorporating the SABV rating into the overall scientific evaluation of the research protocol without asking peer reviewers to provide a separate SABV score. Only one third of participants preferred that the quality of SABV be rated independently but still be factored into the overall evaluation score.

These results were considered in parallel to other stakeholder consultations and independent discussion with decision-makers at the NIH and CIHR. The verdict fell concurrently for both organizations that no specific score would be attributed to the integration of SABV in research protocols. Consensus argued against narrowly defining rules of engagement in applying SABV, as disagreement exists even within the OSSD community on gold standard criteria for assessment. SABV evaluation criteria will be promoted as a guide for peer reviewers when assessing the overall scientific merit of the application, as regards scientific innovation, rigor, reproducibility, and expected impact. The CIHR’s reviewer checklist can be found online [9]. Table 1 illustrates the key questions peer reviewers are encouraged to ask when determining the overall score for the application.

**Table 1:** Questions reviewers should consider when evaluating SABV

Research approach for SABV • Clarity of the research question. • Clarity of rationale for the research approach and methodology. • Appropriateness of the research design. • Appropriateness of the research methods. • Feasibility of the research approach. • Anticipation of difficulties that may be encountered in the research and plans for management. • Quality and appropriateness of SABV. • Justification for a single-sex study. • Evidence that the research question incorporates SABV. • Potential for the research to add value to the current state of knowledge on a given topic that has potential to, but has not yet fully elucidated the impact of sex on biological mechanisms, pathophysiology or translational science. Impact of research incorporating SABV • Potential for a significant contribution to the improvement of women and men’s health, the health of boys and girls, or the health of gender-diverse persons. • Appropriateness and adequacy of the proposed plan for knowledge dissemination and exchange.	

To assist peer reviewers in acquiring competency to evaluate SABV, the CIHR’s Institute of Gender and Health developed an online training module in September 2015 to enable critical appraisal of the appropriate integration, or omission, of SABV in research protocols [10]. This module also aims to fill a gap in knowledge and skills for the 60–80 % of the CIHR biomedical researchers who, during mandatory reporting, indicated that they do not integrate sex or gender into their research designs [11]. The NIH’s Office of Research on Women’s Health similarly held a method workshop and posted an online report to assist scientists debuting the integration of SABV in their research programs [12].

Concerns remain that integrating SABV into research programs may be costly, that resources may not be available, and that duplication of all experimental procedures in both sexes may be onerous and burdensome. If the NIH and CIHR were truly advocating that equal numbers of males and females be included in all studies, then this concern would be valid. However, the NIH and CIHR advocate no such blind change in protocol. Instead, researchers and peer reviewers are being asked to thoughtfully consider whether a single-sex study is justified when research results are to be applied to both sexes. Funding organizations recognize that new, potentially transformative discoveries such as one by a Canadian researcher showing that chronic pain is mediated by different immune cells in males and females will only be brought about by funding studies that attempt to explain sexually dimorphic discrepancies in the epidemiology of disease or response to treatment [13]. By using scientific acumen, researchers are given the freedom to decide if and how SABV may be relevant to their field of study. Will the answer sometimes require researchers to ask for larger budgets for their studies to include equal numbers of males and females? Absolutely. However, if well justified, both the NIH and CIHR believe that the investment in balancing the sexes will be worthwhile.

The topic of statistical power necessary to detect sex differences continues to generate controversy, as does the criticism that policies linked to SABV ignore the contribution of other social determinants of health such as gender, education, and socioeconomic status as risk factors for disease [14–16]. Both the NIH and CIHR recognize that animal models are not the panacea to cure all human suffering. Both organizations simultaneously fund clinical, health systems and population health research to address these questions. With respect to sample size, what is clear is that sample size and power for preclinical studies ultimately depend on the specific question being posed by the researcher. It would be inherently challenging to uniformly implement sample size requirements for SABV across the research spectrum. Discretion will therefore be left to the researcher to justify the sample size required for any given research approach using SABV. Peer reviewers will be asked to weigh in on whether the calculations make sense, in the same way that sample size has been evaluated in the past. No specific score will be recorded for this element.

## Conclusion

The NIH and CIHR intend that peer reviewers should be given the instruction to make sure SABV is accounted for in research design, analysis, and reporting and, if only one sex will be used, that this is well justified. Evaluation of SABV is expected to depend on the best judgment of the reviewers along with other criteria used to assess the scientific excellence of a given proposal. The research question at hand and the specific scientific context should guide appropriate consideration of SABV in a project, with the understanding that transparency is crucial to building a sex-specific evidence base.

## Abbreviations

CIHR: Canadian Institutes of Health Research
NIH: National Institutes of Health
OSSD: Organization for the Study of Sex Differences
SABV: sex as a biological variable
